# The urgency to include palliative care in the response to tuberculosis in Brazil

**DOI:** 10.1590/0102-311XEN164325

**Published:** 2026-02-23

**Authors:** Jorge Luiz Rocha, Paulo Victor de Sousa Viana

**Affiliations:** 1 Escola Nacional de Saúde Pública Sergio Arouca, Fundação Oswaldo Cruz, Rio de Janeiro, Brasil.

## Introduction

Tuberculosis (TB) continues to be one of the leading causes of illness and death due to infectious diseases in the world. In 2024, according to the World Health Organization (WHO), more than 10.7 million people fell ill with TB, resulting in approximately 1.23 million deaths and making it the leading cause of death from a single infectious agent, even surpassing COVID-19 ^1^. In Brazil, 84,308 new cases were reported in 2024, with an incidence coefficient of 39.7 cases per 100,000 inhabitants, and 6,025 deaths were directly attributed to the disease in 2023, which is the highest number recorded in the last 20 years ^2^. Despite being curable, TB still constitutes a challenge for health systems, especially its drug-resistant forms ^3^.

Drug-resistant TB, especially in the multidrug-resistant (MDR-TB) and extensively drug-resistant (XDR-TB) forms, is responsible for aggravating physical and emotional suffering ^4^ ^,^ ^5^. In 2024, teh world recorded around 390,000 new cases of MDR-TB or rifampicin-resistant TB (RR-TB) ^1^. In Brazil, more than 1,200 new cases have been reported. This increasing number is due to the expansion of the rapid molecular test for TB in the Brazilian Unified National Health System (SUS, acronym in Portuguese) ^6^. These forms of the disease require prolonged therapeutic regimens and have multiple side effects, a low success rate, and a strong socioeconomic impact. Recurrent hospitalizations, transportation costs to referral appointments, and fragile family and work ties make the treatment journey burdensome for patients, families, and health teams ^3^ ^,^ ^7^.

TB is recognized as a neglected disease from a clinical and pharmacological standpoint. However, such negligence is not limited to the lack of diagnoses or new drugs; it also involves the negligence of the physical, psychological, and social pain of the affected individuals ^8^. Globally, the field of palliative care has largely ignored TB, which reflects practices originating in the Global North, where the disease is less prevalent ^9^. However, countries in the Global South, such as Brazil, have the opportunity to innovate and develop their own models of palliative care for TB that are appropriate to their epidemiological, cultural, and social realities.

The clinical and social complexity of drug-resistant TB underscores the importance of palliative care as an integral part of the response to the disease. According to the WHO, the aim of palliative is to improve the quality of life of patients and their families facing diseases that threaten the continuity of life through the prevention and relief of suffering. This requires early identification, a proper assessment, and the adequate treatment of pain and other distressing situations of a physical, psychosocial, and spiritual nature. The relevance of this approach for TB resides in the capacity to mitigate the suffering inherent to a burdensome, uncertain therapeutic journey beginning at the time of diagnosis and occurring concomitantly with curative treatment ^10^.

However, the misconception persists that palliative care is only for the end of life ^11^ ^,^ ^12^. This reductionist view prevents individuals with TB, especially in situations of drug resistance or treatment failure, from having access to a person-centered approach focused on dignity, autonomy, and the relief of suffering. Stigma, social exclusion, chronic pain, loneliness, and hopelessness are common occurrences for many patients ^13^ ^,^ ^14^.

The expansion of resistance to drugs used in the treatment of TB is responsible for the existence of programmatically incurable cases, in which there is a lack of therapeutic alternatives among all the options available in the country at the moment. Even with the advent of new drugs recently incorporated into the SUS, such as bedaquiline, delamanid, and pretomanid, there are already reports of simultaneous resistance in some patients ^15^ ^,^ ^16^. When associated with HIV, the scenario becomes even more severe, exerting a strong impact on clinical outcomes ^17^. In many cases, there comes a point at which patients, despite all therapeutic efforts, exhibit no clinical improvement, with the persistence of bacteriological positivity after months of treatment, extensive lung disease, and high-level resistance or progressive clinical deterioration. Patients may even refuse to continue treatment. In such cases, palliative care is no longer optional and becomes imperative ^18^.

The interruption of treatment, although ethically difficult, should be discussed based on clear clinical criteria and with the involvement of the interprofessional team. Patients with an extensive disease that has no surgical or pharmacological options or involves severe clinical deterioration should have immediate access to specialized palliative care. The palliative approach should begin with a comprehensive assessment of the patient’s symptoms and needs, with the planning of care centered on dignity, comfort, and safety ^18^.

Unfortunately, Brazil does not yet have a national protocol for specific palliative care in cases of TB. The absence of such an instrument hinders comprehensive care, leaving patients without adequate therapeutic options and health professionals without clear guidelines to plan strategies to alleviate multidimensional suffering ^19^. Despite the principles of comprehensiveness and universality of the SUS, palliative care in TB is not mentioned in the regulations of the Brazilian Ministry of Health or the guidelines of the different societies of medical specialties.

However, an important step was recently taken with the publication of *Ordinance n. 3.681/2024*, by the Brazilian Ministry of Health, which establishes the Brazilian National Palliative Care Policy (PNCP, acronym in Portuguese) within the scope of the SUS ^20^. This policy constitutes a fundamental regulatory framework for the expansion and institutionalization of palliative care in Brazil, promoting its integration on the different levels of care. Since its creation, however, practical advances have been timid, few teams have been effectively implemented, and TB is still not among the priorities made explicit in the implementation of the policy. This situation underscores the need to broaden the discussion on palliative care beyond oncological diseases, including chronic infectious diseases such as TB.

The PNCP provides for the implementation of palliative care on all levels of the SUS and throughout the country. For this policy to achieve concrete changes, it is essential to overcome historical and conceptual barriers, such as the stigma still surrounding palliative care, which is often misinterpreted as synonymous with therapeutic abandonment. It is necessary to sensitize administrators, healthcare providers, and the population about the strategic role of palliative care as an integral part of person-centered care ^21^. The implementation of this policy will be challenging, with the need to face complex logistical and operational issues, such as providing care for individuals in situations of extreme social vulnerability, including those living on the streets, those deprived of liberty, and those in areas of difficult access. Such groups that are often affected by TB and historically invisible in healthcare networks.

In contrast to this lack of guidelines on palliative care and TB in Brazil, international experience offers ways to address the problem ^22^ ^,^ ^23^ ^,^ ^24^. South Africa is a country with a high burden of TB as well as TB-HIV and has instituted specific palliative care guidelines for this population, including hospitals and specialized referral centers, homecare teams, and spiritual care as well as psychosocial and family support. A survey conducted in the country identified that pain (42.1%), excessive worry (60.5%), a lack of information to enable planning for the future (35.1%), and difficulty in sharing feelings (25.1%) are common in hospitalized patients with TB. Surprisingly, individuals with drug-sensitive TB also report significant distress, demonstrating that palliative care is not restricted to the most severe or untreatable cases. Each person living with TB, regardless of the stage of the disease, is exposed to potential suffering that must be recognized and addressed ^8^.

Brazilian care providers who work on the front line of TB care (physicians, nurses, community health workers, and social workers) are faced with the suffering of their patients on a daily basis and practice, albeit intuitively, fundamental aspects of palliative care: they listen, accept, visit families, and seek to minimize suffering. However, they generally do not receive specific training in palliative care nor have access to opioids for pain control, structured psychological support, or a grief support network ^25^. There is an urgent need for the education and training of teams in basic palliative care skills, the promotion of adequate communication on the prognosis, and the planning of care, including family monitoring and grief support ^12^ ^,^ ^24^.

The development of a national protocol for palliative care in TB should also involve active listening to individuals who have first-hand experience with the disease, especially in its resistant forms. Such individuals have fundamental experience about what it means to become ill, undergo treatment, and often not be cured. Incorporating their reports and perspectives ensures that public policies not only recognize suffering, but are shaped by it, becoming more compassionate, relevant, realistic, and effective ^24^ ^,^ ^26^.

Palliative care in TB needs to be incorporated as an ensured right on all levels of health care. This includes the formation of interprofessional teams, the definition of shared care between primary care and specialized units, management protocols for pain and respiratory symptoms, social and spiritual support, adequate communication about the prognosis, and the planning of care. It is also necessary to differentiate the general palliative approach that all healthcare providers should take from specialized care performed by teams with specific training and interdisciplinary action ^18^ ^,^ ^24^ ^,^ ^27^.

A national protocol for palliative care for TB should envisage the integration of TB services with teams specialized in palliative care, the use of quality of life and suffering indicators as part of the clinical assessment, and the incorporation of the principles of dignity, autonomy, and person-centered care. Such a protocol should encompass the physical, psychological, social, and spiritual dimensions of the illness ^24^ and consider patients with TB as being worthy of care and not merely a case of notification. Public policy must also contemplate the post-mortem impact. Families that lose loved ones amidst suffering, without support or guidance, remain marked by lasting emotional trauma. Unassisted grief perpetuates suffering and compromises the bond between communities and healthcare services ^18^. Thus, palliative care is also a public health strategy capable of reducing collective suffering and strengthening social trust in health institutions, as demonstrated in a randomized trial conducted in Uganda, in which a nurse-led model resulted in significant improvements in both quality of life and the relationship between patients and healthcare services ^28^.

To promote a more compassionate, efficient, equitable response to the suffering associated with TB, especially in its resistant forms, the following operational recommendations are presented for the incorporation of palliative care into the SUS ^19^ ^,^ ^24^:

(1) Develop and implement a national protocol for palliative care in TB based on evidence, international experiences, and social participation.

(2) Include training in palliative care in the curricula of continuing education for healthcare providers who work in the TB care network.

(3) Implant specialized palliative care teams and units at referral centers and in regions with a high TB burden.

(4) Ensure the continuous supply of essential medications for pain and the management of other symptoms in all regions.

(5) Incorporate indicators of suffering and quality of life into health information systems.

(6) Ensure psychosocial and spiritual support for patients and their families, with a focus on listening, acceptance, and respect for diversity.

(7) Adapt care facilities with adequate infrastructure for the dignified reception of patients with incurable TB, with a focus on nutrition, leisure, support, and the prevention of transmission.

(8) Invest in qualitative and quantitative assessment studies on suffering, palliative care, and TB outcomes.

Scientific knowledge, international experience, and the legal framework in force in Brazil, as represented by the PNCP ^20^, provide the necessary foundation for the full integration of palliative care in the response to TB. It is imperative for institutions and public administrators to demonstrate the sensitivity and political commitment necessary to ensure this right. It is unacceptable to consider therapeutic abandonment and suffering as a natural course for individuals for whom a cure is no longer a possibility. It must be ensured that, when there is no longer a chance of a cure, there is always the assurance of dignified care.

## Final considerations

TB is indeed a curable disease, but this does not apply to all patients. For those who are not cured, the healthcare system must offer listening, comfort, and dignity in addition to technical protocols. The Brazilian response to TB needs to evolve to include comprehensive, compassionate care in a structured way. The creation of a national palliative care protocol for TB is a decisive step towards the humanization of care, equity in health, and social justice. It is essential for the suffering of these individuals to be made visible. Above all, humans and their inalienable right not to suffer in silence must remain at the center of any health policy.
