# Supplementary material for: The urgency to include palliative care in the response to tuberculosis in Brazil
Source: Cad Saude Publica. 2026 Feb 23;42:e00164325. doi: 10.1590/0102-311XEN164325 (PMC12928555; doi:10.1590/0102-311XEN164325)
Supplement: Translation

# Translation: A urgência de integrar os cuidados paliativos na resposta à tuberculose no Brasil

**DOI:** 10.1590/0102-311XPT164325

**Authors:** Jorge Luiz Rocha, Paulo Victor de Sousa Viana

**Affiliations:** 1 Escola Nacional de Saúde Pública Sergio Arouca, Fundação Oswaldo Cruz, Rio de Janeiro, Brasil.

## Introdução

A tuberculose (TB) continua sendo uma das principais causas de adoecimento e morte por doença infecciosa no mundo. Em 2024, segundo a Organização Mundial da Saúde (OMS), mais de 10,7 milhões pessoas adoeceram por TB, resultando em aproximadamente 1,23 milhão de mortes, colocando a TB como a principal causa de morte por um único agente infeccioso no mundo, superando a COVID-19 ^1^. No Brasil, em 2024, foram notificados 84.308 casos novos de TB, com um coeficiente de incidência de 39,7 casos por 100 mil habitantes e 6.025 óbitos atribuídos diretamente à doença em 2023, o maior número registrado nos últimos 20 anos ^2^. Apesar de curável, a TB ainda representa um desafio estruturante para os sistemas de saúde, principalmente em suas formas resistentes aos medicamentos ^3^.

A TB drogarresistente (TB-DR), especialmente nas formas multidrogarresistente (TB-MDR) e extensivamente resistente (TB-XDR), é responsável por agravar o sofrimento físico e emocional ^4^ ^,^ ^5^. Em 2024, o mundo registrou cerca de 390 mil casos novos de TB-MDR ou resistente à rifampicina (TB-RR) ^1^. No Brasil, mais de 1.200 casos novos foram notificados, número crescente em razão da ampliação do teste rápido molecular (TRM-TB) no Sistema Único de Saúde (SUS) ^6^. Essas formas da doença exigem esquemas terapêuticos prolongados, com múltiplos efeitos colaterais, baixa taxa de sucesso e forte impacto socioeconômico. As internações recorrentes, os custos com transporte para as consultas nas referências e a fragilidade de vínculos familiares e laborais tornam a jornada do tratamento penosa aos pacientes, famílias e para as equipes de saúde ^3^ ^,^ ^7^.

Apesar de ser reconhecida como uma doença negligenciada sob a perspectiva clínica e farmacológica, a TB também é negligenciada na dimensão do sofrimento humano. Essa negligência não se limita à falta de diagnóstico ou novas drogas, mas à invisibilidade da dor física, psicológica e social das pessoas acometidas ^8^. Globalmente, o campo dos cuidados paliativos tem, em grande parte, ignorada a TB, o reflexo de um campo do conhecimento e de práticas oriundos do Norte Global, onde a doença é menos incidente ^9^. Nesse cenário, os países do Sul Global, como o Brasil, têm a oportunidade de inovar e desenvolver modelos próprios de cuidados paliativos voltados à TB, adequados às suas realidades epidemiológicas, culturais e sociais.

A complexidade clínica e social da TB-DR evidencia a importância dos cuidados paliativos como parte integrante da resposta à doença. De acordo com a OMS, cuidados paliativos consistem em uma abordagem que visa melhorar a qualidade de vida de pacientes e suas famílias diante de doenças que ameaçam a continuidade da vida, por meio da prevenção e alívio do sofrimento. Para tal, requer a identificação precoce, avaliação e tratamento impecável da dor e outras situações angustiantes de natureza física, psicossocial e espiritual. A relevância dessa abordagem para a TB reside na sua capacidade de atuar desde o diagnóstico, de forma concomitante ao tratamento curativo, mitigando o sofrimento inerente a uma jornada terapêutica penosa e de incertezas ^10^.

No entanto, persiste o equívoco que associa cuidados paliativos apenas ao fim da vida ^11^ ^,^ ^12^. Essa visão reducionista impede que pessoas com TB, especialmente em situações de resistência medicamentosa ou falha terapêutica, tenham acesso a uma abordagem centrada na pessoa, com foco no alívio do sofrimento, na dignidade e na autonomia. O estigma, a exclusão social, a dor crônica, a solidão e a desesperança são realidades cotidianas para muitos pacientes ^13^ ^,^ ^14^.

A expansão da resistência às drogas utilizadas no tratamento da TB é responsável pela existência de casos programaticamente incuráveis, para os quais faltam alternativas terapêuticas dentre todas as opções disponíveis no país no momento. Mesmo com o advento de medicamentos novos, recentemente incorporados ao SUS, como bedaquilina, delamanida e pretomanida, já há relatos de resistência simultânea em alguns pacientes ^15^ ^,^ ^16^. Quando associado ao HIV, o cenário se torna mais grave, impactando fortemente os desfechos clínicos ^17^. Em muitos casos, mesmo com todos os esforços terapêuticos, chega-se a um ponto em que o paciente não apresenta melhora clínica, seja por persistência de positividade bacteriológica após meses de tratamento, doença pulmonar extensa, resistência de alto nível, deterioração clínica progressiva ou recusa, informada pelo paciente, à continuidade do tratamento. Nesses casos, os cuidados paliativos deixam de ser opcionais e tornam-se imperativos ^18^.

A interrupção do tratamento, ainda que eticamente difícil, deve ser discutida com base em critérios clínicos claros e com envolvimento da equipe interprofissional. Os pacientes com doença extensa, sem opções cirúrgicas ou farmacológicas, ou com grave deterioração clínica, devem ter acesso imediato a cuidados paliativos especializados. A abordagem paliativa deve ser iniciada com uma avaliação abrangente dos sintomas e necessidades do paciente, construindo um plano de cuidado centrado na dignidade, no conforto e na segurança ^18^.

Infelizmente, o Brasil ainda não possui um protocolo nacional de cuidados paliativos específicos para a TB. A ausência desse instrumento dificulta o cuidado integral, deixando pacientes sem alternativas terapêuticas adequadas e profissionais de saúde sem diretrizes claras para planejar estratégias de alívio do sofrimento multidimensional ^19^. Apesar dos princípios de integralidade e universalidade do SUS, os cuidados paliativos em TB seguem invisíveis nas normativas do Ministério da Saúde e nas diretrizes das sociedades das especialidades médicas.

Entretanto, um passo importante foi dado recentemente com a publicação da *Portaria nº 3.681/2024*, do Ministério da Saúde, que institui a Política Nacional de Cuidados Paliativos (PNCP) no âmbito do SUS ^20^. Essa política representa um marco regulatório fundamental para a expansão e institucionalização dos cuidados paliativos no Brasil, promovendo sua integração aos diferentes níveis de atenção. Contudo, desde sua criação, os avanços práticos têm sido tímidos, poucas equipes foram efetivamente implantadas e a TB ainda não figura entre as prioridades explicitadas na implementação política. Essa realidade reforça a necessidade de expandir o debate sobre cuidados paliativos para além das doenças oncológicas, incluindo agravos infecciosos crônicos como a TB.

A PNCP prevê a implementação de cuidados paliativos por meio de articulação entre equipes matriciais e assistenciais, em todos os níveis de atenção do SUS e em todo o país. Para que essa política se reflita em mudanças concretas, é fundamental superar de barreiras históricas e conceituais, por exemplo, a necessidade de reverter o estigma ainda associado aos cuidados paliativos, frequentemente mal interpretados como sinônimos de abandono terapêutico. É preciso sensibilizar gestores, profissionais de saúde e a população sobre o papel estratégico dos cuidados paliativos como parte integrante da atenção à saúde centrada na pessoa ^21^. Além disso, a efetivação dessa política será desafiadora, exigindo o enfrentamento de questões logísticas e operacionais complexas, como o cuidado a pessoas em situação de vulnerabilidade social extrema, incluindo aqueles em situação de rua, os privados de liberdade ou em territórios de difícil acesso, grupos estes frequentemente acometidos pela TB e historicamente invisibilizados nas redes de atenção à saúde.

Em contraste com essa ausência de diretrizes em cuidados paliativos e TB no Brasil, a experiência internacional aponta caminhos ^22^ ^,^ ^23^ ^,^ ^24^. A África do Sul, país de alta carga de TB e TB-HIV, instituiu diretrizes especificas de cuidados paliativos para pessoas com TB, incluindo hospitais e centros de referência especializados, equipes domiciliares, cuidados espirituais e apoio psicossocial e familiar. Em um inquérito realizado na África do Sul, identificou-se que sintomas como dor (42,1%), preocupação excessiva (60,5%), falta de informações para planejar o futuro (35,1%) e a dificuldade de compartilhar sentimentos (25,1%) são comuns em pacientes com TB hospitalizados. E, de forma surpreendente, pessoas com TB sensível também relataram sofrimento significativo, reforçando que os cuidados paliativos não se restringem apenas aos casos mais graves ou intratáveis. Cada pessoa vivendo com TB, independentemente do estágio da doença, carrega um potencial de sofrimento que deve ser reconhecido e acolhido ^8^.

Os profissionais brasileiros que atuam na linha de frente do cuidado da TB, médicos, enfermeiros, agentes comunitários de saúde e assistentes sociais já enfrentam o sofrimento de seus pacientes diariamente e já praticam, ainda que de forma intuitiva, aspectos fundamentais dos cuidados paliativos: escutam, acolhem, visitam famílias, buscam minimizar sofrimentos. No entanto, em geral, não recebem formação específica em cuidados paliativos, tampouco contam com acesso a opioides para controle da dor, suporte psicológico estruturado ou rede de apoio ao luto ^25^. A formação e capacitação de equipes em competências básicas de cuidados paliativos, e a promoção de uma comunicação adequada sobre prognósticos e planejamento do cuidado, incluindo o acompanhamento familiar e o suporte ao luto, são urgentes ^12^ ^,^ ^24^.

A elaboração de um protocolo nacional de cuidados paliativos em TB deve, ainda, envolver a escuta ativa de pessoas que conviveram com a doença, especialmente nas formas resistentes. Esses indivíduos carregam vivências fundamentais sobre o que significa adoecer, tratar e, muitas vezes, não ser curado. Incorporar seus relatos e perspectivas garante que as políticas públicas não apenas reconheçam o sofrimento, mas sejam moldadas por ele, tornando-se mais humanas, relevantes, realistas e eficazes ^24^ ^,^ ^26^.

O cuidado paliativo em TB precisa ser incorporado como um direito garantido em todos os níveis de atenção à saúde. Isso inclui a formação de equipes interprofissionais, definição de linhas de cuidado compartilhado entre atenção primária e unidades especializadas, protocolos de manejo da dor e de sintomas respiratórios, suporte social e espiritual, comunicação adequada sobre prognóstico e planejamento de cuidados. Também é necessário diferenciar a abordagem paliativa geral que todos os profissionais devem aplicar dos cuidados especializados, realizados por equipes com formação específica e atuação interdisciplinar ^18^ ^,^ ^24^ ^,^ ^27^.

Um protocolo nacional de cuidados paliativos para TB deve prever, de forma clara, a integração dos serviços de TB com equipes especializadas em cuidados paliativos, a utilização de indicadores de qualidade de vida e de sofrimento como parte da avaliação clínica, e a incorporação dos princípios de dignidade, autonomia e cuidado centrado na pessoa. Deve contemplar as dimensões físicas, psicológicas, sociais e espirituais do adoecimento ^24^. E, acima de tudo, deve colocar o paciente com TB como sujeito pleno de cuidado, não apenas como caso de notificação. A política pública deve contemplar também o impacto pós-morte. As famílias perdem entes queridos em sofrimento, sem apoio, sem orientação, permanecem marcadas por traumas e sequelas emocionais e duradouras. O luto não assistido perpetua o sofrimento e compromete o vínculo entre comunidades e os serviços de saúde ^18^. Os cuidados paliativos são, também, uma estratégia de saúde pública capaz de reduzir o sofrimento coletivo e fortalecer a confiança social nas instituições de saúde, como demonstrado no estudo randomizado conduzido em Uganda, onde um modelo liderado por enfermeiras resultou em melhora significativa da qualidade de vida e da relação entre pacientes e serviços de saúde ^28^.

A fim de promover uma resposta mais humana, eficiente e equitativa ao sofrimento associado à TB, especialmente nas suas formas resistentes, são apresentadas as seguintes recomendações operacionais para a incorporação dos cuidados paliativos no SUS ^19^ ^,^ ^24^:

(1) Desenvolvimento de protocolo nacional: elaborar e implementar um protocolo nacional de cuidados paliativos em TB, com base em evidências, experiências internacionais e participação social.

(2) Capacitação profissional: incluir formação em cuidados paliativos nos currículos da educação permanente dos profissionais que atuam na rede de atenção à TB.

(3) Serviços especializados: implantar equipes e unidades de cuidados paliativos em centros de referência e em regiões de alta carga de TB.

(4) Acesso a medicamentos: assegurar o fornecimento contínuo de medicamentos essenciais para o controle da dor e de outros sintomas em todas as regiões.

(5) Indicadores de sofrimento e qualidade de vida: incorporar indicadores sensíveis ao sofrimento nos sistemas de informação em saúde.

(6) Apoio psicossocial e espiritual: garantir apoio psicossocial e espiritual para pacientes e suas famílias, com enfoque na escuta, acolhimento e respeito à diversidade.

(7) Infraestrutura adequada: adaptar espaços físicos de cuidado para acolhimento digno de pacientes com TB incurável, com foco em nutrição, lazer, apoio e prevenção da transmissão.

(8) Pesquisa e avaliação: investir em estudos qualitativos e quantitativos sobre sofrimento, cuidados paliativos e desfechos em TB.

O conhecimento científico, a experiência internacional e o arcabouço legal vigente no Brasil, representado pela PNCP ^20^, fornecem o fundamento necessário para a plena integração dos cuidados paliativos na resposta à TB. Torna-se imperativo que as instituições e a gestão pública demonstrem a sensibilidade e o compromisso político necessários para concretizar esse direito. É inaceitável naturalizar o abandono terapêutico e o sofrimento das pessoas para as quais a cura não é mais uma possibilidade, devendo-se assegurar que onde não há mais chance de cura, haja sempre a garantia de cuidado digno.

## Considerações finais

A TB é, de fato, uma doença curável, mas essa realidade não se aplica a todos os pacientes. Para aqueles que não alcançam a cura, o sistema de saúde deve oferecer, além dos protocolos técnicos, a presença, a escuta, o conforto e a dignidade. A resposta brasileira à TB precisa evoluir para incluir, de maneira estruturada, o cuidado integral e compassivo. A construção de um protocolo nacional de cuidados paliativos para TB é um passo decisivo rumo à humanização do cuidado, à equidade em saúde e à justiça social. É fundamental que o sofrimento dessas pessoas seja visibilizado. Acima de tudo, o ser humano e seu direito inalienável de não sofrer em silêncio devem permanecer no centro de qualquer política de saúde.
